# Effects of Proton Pump Inhibitor Administration and Intake of a Combination of Yogurt and Galactooligosaccharides on Bone and Mineral Metabolism in Rats

**DOI:** 10.3390/nu8100653

**Published:** 2016-10-21

**Authors:** Satoshi Takasugi, Miho Shioyama, Masami Kitade, Masashi Nagata, Taketo Yamaji

**Affiliations:** Food Science Research Laboratories, Division of Research and Development, Meiji Co., Ltd., Odawara 250-0862, Japan; miho.shioyama@meiji.com (M.S.); masami.kitade@meiji.com (M.K.); masashi.nagata@meiji.com (M.N.); taketo.yamaji@meiji.com (T.Y.)

**Keywords:** omeprazole, proton pump inhibitor, bone mineral density, FGF23, calcitriol, phosphorus

## Abstract

The aim of this study was to investigate the effects of proton pump inhibitor (PPI), the most potent acid-suppressing drug, administration and intake of a combination of yogurt and galactooligosaccharides (YG) on bone and mineral metabolism in adult rats. Twelve-week-old male Wistar rats were divided into three groups: a control group fed the control diet with vehicle administration, a PPI group fed the control diet with PPI administration and a YG + PPI group fed the YG diet with PPI administration. All of the groups received their respective experimental diets and daily subcutaneous injection of the vehicle or PPI for 12 weeks. The PPI group showed significantly lower bone mineral density (BMD) of the femur and the lumbar vertebrae and serum fibroblast growth factor 23 (FGF23) and significantly higher phosphorus absorption and serum 1,25-dihydroxyvitamin D (1,25(OH)2D) than the control group, although PPI did not affect calcium absorption. The PPI + YG group showed significantly higher BMD and serum FGF23 and significantly lower phosphorus absorption and serum 1,25(OH)2D than the PPI group. Furthermore, the PPI + YG group showed higher calcium absorption than the control group. These results suggest that although PPI administration did not affect calcium absorption, it adversely affected BMD and influenced phosphorus metabolism in adult rats. Furthermore, the YG diet beneficially affected BMD and attenuated the effects of PPI administration on phosphorus metabolism.

## 1. Introduction

Some drugs have been reported to interact with the availability of nutrients. Proton pump inhibitors (PPIs), the most potent acid-suppressing drugs, are among the most commonly-used drugs worldwide and are generally considered safe [1]. In contrast, some epidemiological studies have shown that the use of PPI is associated with an increased risk of fracture [2,3,4,5]. The assumed mechanism is that PPI use decreases calcium absorption, subsequently leading to lower bone mass [6]. In fact, we have previously reported that PPI administration decreased calcium absorption, which resulted in decreased bone mineral density (BMD) and bone strength in weaning rats [7,8].

The usefulness of cow milk [9,10] or dairy products, including yogurt and cheese [11], for improving BMD has been reported. Galactooligosaccharides (GOS) are low-digestible carbohydrates, are resistant to small intestinal digestion and ferment in the large intestine. GOS fermentation in the large intestine facilitated the absorption of calcium [12,13,14] and increased BMD in weaning rats [15] and ovariectomized rats [16]. We have reported that a combination of dairy product fermented by lactobacilli and GOS improved calcium absorption in an additive manner and increased bone strength in weaning rats receiving PPI administration [8].

1,25-dihydroxyvitamin D (1,25(OH)2D) is an important regulator of calcium and phosphorus homeostasis and is known to stimulate active the pathway of calcium [17] and phosphorus absorption [18]. In contrast, we have previously shown that PPI administration decreased calcium absorption and did not affect phosphorus absorption in weaning rats, although it increased 1,25(OH)_2_D levels [7], which suggests that high 1,25(OH)_2_D levels induced by PPI administration did not compensate for the decreased calcium absorption in weaning rats. Calcium absorption occurs by both an active saturable transcellular pathway and a passive non-saturable paracellular pathway [17,19]. During the weaning period in rats, the mechanisms of calcium absorption change from a paracellular pathway, which is insensitive to vitamin D, to a combination of a paracellular pathway and a transcellular vitamin D-dependent pathway [20]. Therefore, the previous study design may have been unusual in that weaning rats were used to investigate the effects of PPI administration and the intake of a combination of dairy product fermented by lactobacilli and GOS on calcium metabolism and subsequent changes of bone metabolism. The aim of this study was to investigate the effects of PPI administration and the intake of a combination of yogurt and GOS (YG) on bone and mineral metabolism in adult rats.

## 2. Experimental Section

### 2.1. Diets

Yogurt was produced as follows: Skim milk was cultured with *Lactobacillus bulgaricus* and *Streptococcus thermophilus* and subsequently lyophilized. The lyophilized yogurt was sterilized by 10-kGy electron beam irradiation.

We employed AIN-93M as the control diet (0.5% calcium and 0.3% phosphorus) (Table 1). For the YG diet, a part of the casein was replaced with lyophilized yogurt. GOS was added instead of sucrose at the level of 5.0% of the diets. Calcium, phosphorus and crude protein were adjusted to the same level between the experimental diets. Crude protein in the diets was calculated as total Kjeldahl nitrogen × 6.38. The GOS ingredient (Cup-Oligo) contained 73% GOS and was kindly provided by Nissin Sugar manufacturing Co., Ltd. (Tokyo, Japan).

### 2.2. Animals

Twenty-four, 11-week-old male Wistar rats were purchased from Japan SLC (Shizuoka, Japan) and cared for in accordance with the guidelines for animal studies of the Kyoto Institute of Nutrition and Pathology and Law No. 105 and Notification No. 6 of the Government of Japan (Ethic approval code: 10007MN-N). The rats were individually housed in stainless steel metabolic cages in a light-controlled room (12-h light/dark cycle) at ambient temperature (25 °C) throughout the study. After a 1-week adaptation, rats were divided into three body weight-matched groups of eight rats each: a control group fed the control diet with vehicle administration, a PPI group fed the control diet with PPI administration, and a PPI + YG group fed the YG diet with PPI administration. All of the groups were fed their respective experimental diets only during the dark phase for 12 weeks. The control and PPI groups were pair-fed to the PPI + YG group. The PPI and PPI + YG groups were subcutaneously injected with omeprazole sodium (PPI) (Omepral Injection 20; AstraZeneka, Osaka, Japan) at a dose of 20 mg/kg (4 mg/mL in physiological saline) every day, 1 h before the dark phase, when rats were fed their respective diets during the experimental period. In the preliminary study, we confirmed that the pH values of cumulative gastric juice in adult rats receiving PPI at this dose were significantly higher at 2–5 h (7.0 ± 0.2) and 9–12 h (2.5 ± 0.3) after the administration compared with the basal level (1.8 ± 0.2) [21]. The control group was subcutaneously injected with physiological saline in the same manner. All of the groups were allowed free access to demineralized water during the experimental period.

All feces of each rat were collected for 3 consecutive days at the 4th and 12th weeks of the experimental period. The fecal samples were used to determine calcium and phosphorus concentrations. Tetracycline (20 mg/kg) and calcein (20 mg/kg) were subcutaneously injected for bone labeling 6 and 2 days before sacrifice. At the end of the experimental period, blood samples were obtained from the abdominal aorta under pentobarbital anesthesia. The serum samples were separated by centrifugation at 3000 rpm for 15 min at 4 °C and stored at −80 °C until analysis. After sacrifice, each rat’s right femur, tibia, lumbar vertebrae (LV) and whole caecum were excised. The right femurs and LV were stored in a 70% ethanol solution (Wako Pure Chemical Industries, Osaka, Japan) for X-ray computed tomography (CT) analysis. The right tibias were stored in 70% ethanol solution for bone histomorphometry. The whole caecal weight was measured.

### 2.3. BMD by X-ray CT Analysis

The whole right femur and the 6th and 7th LV were scanned at 1-mm intervals using the LaTheta (LCT-100M) experimental animal CT system (ALOKA, Tokyo, Japan). Contiguous 0.6-mm slices for the whole femur and 0.7-mm slices for the 6th and 7th LV were used for quantitative assessment. Total BMD, cortical BMD and trabecular BMD of the whole femur and the LV were calculated using LaTheta software (Version 1.31).

### 2.4. Dynamic Bone Histomorphometry

Dynamic bone histomorphometry was performed with the trabecular bone at the right proximal tibial metaphysis at the Ito Bone Histomorphometry Institute (Niigata, Japan). Briefly, after the removal of soft tissues, the proximal part of the right tibia was subjected to Villanueva bone staining for 4 days without decalcifying treatment, dehydrated with increasing concentrations of ethanol and embedded in methyl methacrylate. The proximal tibia was sliced in the frontal plane (5-μm thickness) with a microtome (Leica, Germany). Regions of the secondary cancellous bone located 945–1890 μm distal to the growth plate cartilage of the proximal tibia were selected. The measured area was 2.38 mm^2^. The bone formation rate/bone surface (BFR/BS, mm^3^/mm^2^/year) and bone resorption rate (BRs.R, µm^2^/µm^2^/year) were calculated as previously described [22,23]. The nomenclature and units used were in accordance with the American Society for Bone and Mineral Research (ASBMR) Histomorphometry Nomenclature Committee [22].

### 2.5. Calcium and Phosphorus Absorption

Diet and feces samples were mineralized in trace element-grade concentrated nitric acid (Wako Pure Chemical Industries, Osaka, Japan) using a microwave system (Multiwave 3000; Perkin Elmer, Tokyo, Japan) and analyzed for calcium and phosphorus by inductively-coupled plasma spectroscopy (ICP-S7500; Shimadzu, Kyoto, Japan). The amount of apparent calcium absorption (mg/3 days) was calculated as (calcium intake-fecal calcium). The amount of apparent phosphorus absorption was calculated in the same manner.

### 2.6. Biochemical Analysis

Serum levels of intact parathyroid hormone (PTH), fibroblast growth factor 23 (FGF23) and 1,25-dihydroxyvitamin D (1,25(OH)2D) were assayed using the Rat BioActive Intact PTH ELISA kit (Immnunotopics, San Clementre, CA, USA), the FGF23 ELISA kit (Kainos, Tokyo, Japan) and the 1,25-Dihydroxy Vitamin D EIA kit (Immunodiagnostic Systems, Nordic a/s, Herlev, Denmark), respectively. Serum calcium and phosphorus levels were colorimetrically determined by commercial kits (Wako Pure Chemical Industries, Osaka, Japan).

### 2.7. Statistics

Data are expressed as the mean values with their standard errors. Bartlett’s test was performed to determine the homogeneity of variances. If variances were homogeneous, treatment effects were analyzed by one-way ANOVA followed by Fisher’s least significant difference test. If variances were heterogeneous, the Kruskal–Wallis test followed by the Steel–Dwass multiple comparison test were used. Differences were considered significant at *p* < 0.05. All statistical analyses were performed with the Ekuseru-Toukei 2012 software (Social Survey Research Information Co., Ltd., Tokyo, Japan). A correlation coefficient between BMD of femur and serum FGF23 or 1,25(OH)_2_D levels was calculated by the least squares method using Microsoft Excel 2010 (Microsoft, Tokyo, Japan).

## 3. Results

### 3.1. Body Weight and Whole Caecal Weight

Body weight at the termination of the experiment was 340.6 ± 5.2 g in the control group, 347.3 ± 4.2 g in the PPI group and 348.6 ± 5.5 g in the PPI + YG group. No significant difference was observed in the final body weight among the groups. The means (±SE) of whole caecal weight in the control, PPI and PPI + YG groups were 3.06 ± 0.18, 3.52 ± 0.15 and 8.66 ± 0.60 g, respectively. The whole caecal weight was significantly higher in the PPI + YG group than in the other groups.

### 3.2. BMD of the Femur and the LV

**Figure 1:**
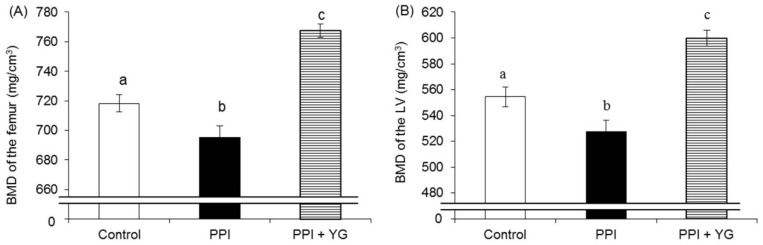

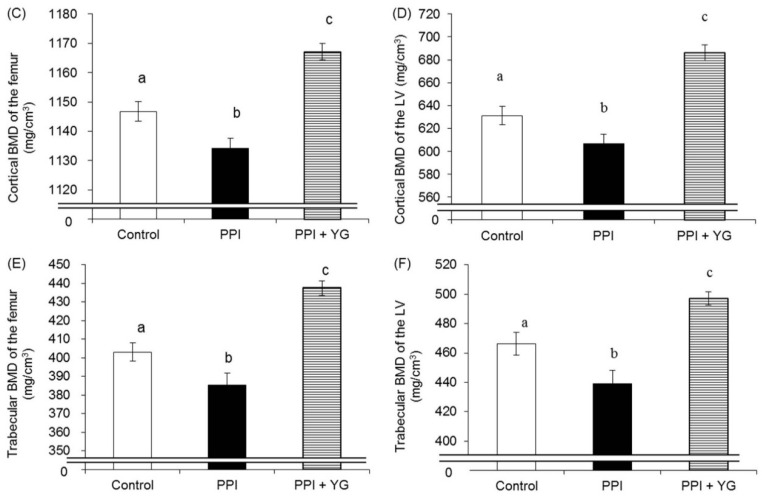
Effects of proton pump inhibitor (PPI) administration and intake of a combination of yogurt and galactooligosaccharides (YG) on bone mineral density (BMD) in adult rats. (**A**) BMD of the femur; (**B**) BMD of the lumbar vertebrae (LV); (**C**) Cortical BMD of the femur; (**D**) Cortical BMD of the LV; (**E**) Trabecular BMD of the femur; (**F**) Trabecular BMD of the LV. Values are presented as the mean ± SE. a, b, c: bars with different letters are significantly different (*p* < 0.05).

Total BMD, cortical BMD and trabecular BMD of the femur and the LV were significantly lower in the PPI group than in the control group and significantly higher in the PPI + YG group than in the other groups (Figure 1A–F).

### 3.3. Dynamic Bone Histomorphometry

**Figure 2:**
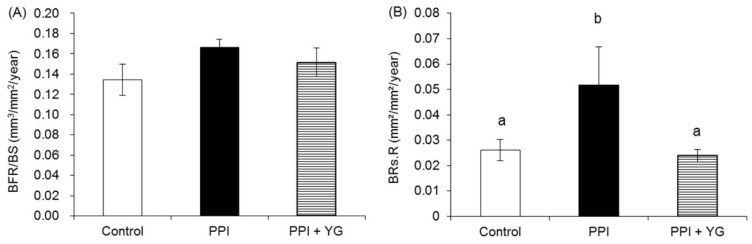
Effects of proton pump inhibitor (PPI) administration and the intake of a combination of yogurt and galactooligosaccharides (YG) on dynamic bone histomorphometry of proximal tibial metaphysis in adult rats. (**A**) Bone formation rate/bone surface (BFR/BS); (**B**) Bone resorption rate (BRs.R). Values are presented as the mean ± SE. a, b: bars with different letters are significantly different (*p* < 0.05).

BFR/BS did not differ among the groups (Figure 2A). BRs.R was significantly higher in the PPI group than in the other groups (Figure 2B).

### 3.4. Biochemical Analysis

**Figure 3:**
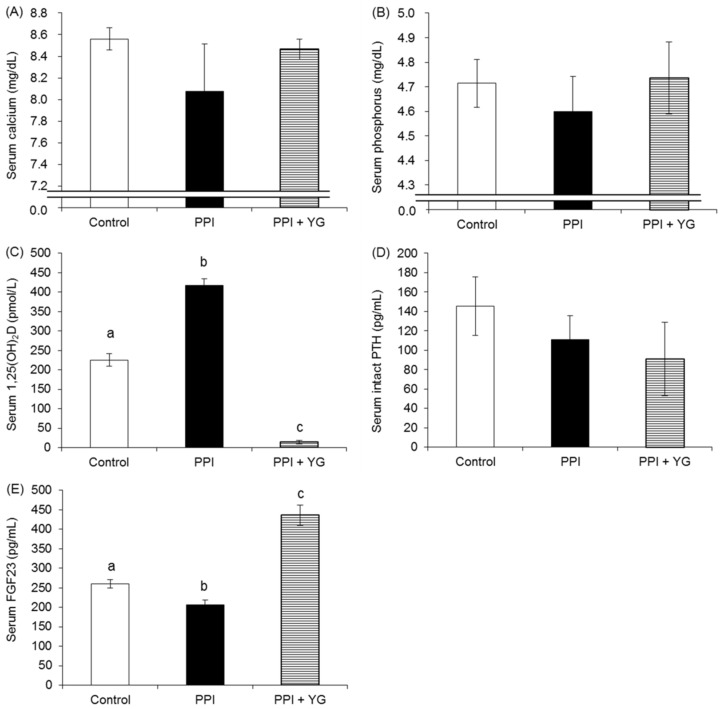
Effects of proton pump inhibitor (PPI) administration and the intake of a combination of yogurt and galactooligosaccharides (YG) on biochemical factors in adult rats. (**A**) Serum calcium levels; (**B**) Serum phosphorus levels; (**C**) Serum 1,25(OH)_2_D levels; (**D**) Serum intact parathyroid hormone (PTH) levels; (**E**) Serum FGF23 levels. Values are presented as the mean ± SE. a, b, c: bars with different letters are significantly different (*p* < 0.05).

There was no significant difference in the serum calcium (Figure 3A) and phosphorus levels (Figure 3B). Serum 1,25(OH)_2_D levels were significantly higher in the PPI group than in the control group and significantly lower in the PPI + YG group than in the other groups. Serum intact PTH levels did not differ among the groups (Figure 3D). Serum FGF23 levels were significantly lower in the PPI group than in the control group and significantly higher in the PPI + YG groups than in the other groups (Figure 3E). There were significant correlations between BMD of the whole femur and both serum FGF23 levels (*r* = 0.8180, *p* < 0.0001) and serum 1,25(OH)_2_D levels (*r* = −0.8881, *p* < 0.0001).

### 3.5. Calcium and Phosphorus Absorption

**Figure 4:**
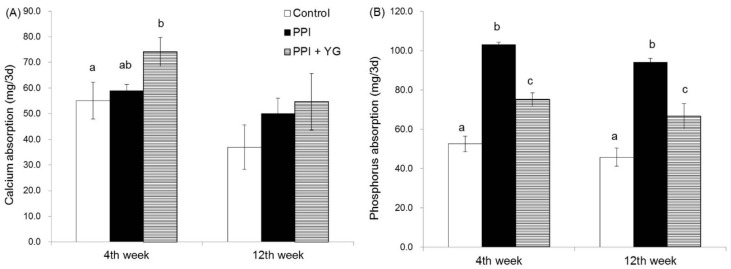
Effects of proton pump inhibitor (PPI) administration and the intake of a combination of yogurt and galactooligosaccharides (YG) on calcium and phosphorus absorption in adult rats. (**A**) Calcium absorption; (**B**) Phosphorus absorption. Values are presented as the mean ± SE; a, b, c: bars with different letters are significantly different (*p* < 0.05).

There was no significant difference in the calcium absorption at the fourth and 12th week between the control group and the PPI group; however, calcium absorption at the fourth week was significantly higher in the PPI + YG group than in the control group and tended to be higher (*p* = 0.0635) in the PPI + YG group than in the PPI group (Figure 4A). Phosphorus absorption at the fourth and 12th week was significantly higher in the PPI group than in the other groups (Figure 4B). In contrast, phosphorus absorption at the fourth and 12th week was significantly lower in the PPI + YG group than in the PPI group.

## 4. Discussion

The present study aimed to investigate the effects of PPI administration and the intake of the YG diet on bone and mineral metabolism in adult rats. The present study showed that PPI administration decreased BMD in adult rats, which was improved by the YG diet. Some studies have showed that the use of PPI was associated with lower BMD in older individuals [24] and maintenance hemodialysis patients [25], which is consistent with our results. Bone dynamic histomorphometric parameters showed that PPI administration increased bone resorption, which was attenuated by the YG diet. In contrast, both PPI administration and the YG diet did not influence the bone formation rate. These results may explain the changes in BMD.

We found that PPI administration did not alter calcium absorption in adult rats, while the previous studies have shown that it decreased calcium absorption in weaning rats [7,8], suggesting that the effects of PPI administration on calcium absorption depend on age. Calcium absorption occurs by both an active saturable transcellular pathway and a passive non-saturable paracellular pathway, and 1,25(OH)_2_D is the major stimulator of an active transcellular pathway [17,19]. During the weaning period in the rat, the mechanisms of calcium absorption change from a paracellular pathway, which is insensitive to vitamin D, to a combination of a paracellular pathway and a transcellular vitamin D-dependent pathway [20], which suggests that the contribution of the vitamin D-dependent pathway to calcium absorption is larger in adult rats than in weaning rats. In the present study, increased 1,25(OH)_2_D may largely contribute to the maintenance of calcium absorption in the PPI-administered rats.

Interestingly, PPI administration remarkably increased phosphorus absorption in adult rats. Phosphorus is transported into intestinal epithelial cells by cotransport with sodium, and the expression is enhanced by 1,25(OH)_2_D [26]. The increased 1,25(OH)_2_D levels in PPI-administered rats may lead to the increase in phosphorus absorption. The production of 1,25(OH)_2_D is enhanced by PTH and inhibited by FGF23 [26]. We showed that PPI administration decreased serum FGF23 levels, while it did not affect serum PTH levels. Therefore, the decrease in FGF23 levels may be responsible for the increase in serum 1,25(OH)_2_D levels. FGF23 is upregulated by PTH [27,28] and 1,25(OH)_2_D [29] and downregulated by hypocalcemia [30]. In contrast, the present study showed that PPI administration did not significantly affect serum PTH and calcium levels, and it increased serum 1,25(OH)_2_D levels, suggesting that these factors were unlikely to be responsible for the decrease in FGF23 levels in PPI-administered rats. Further studies are required to elucidate the mechanism by which PPI administration influenced serum FGF23 and 1,25(OH)_2_D levels.

The present study showed that the YG diet increased calcium absorption in adult rats, which was consistent with the previous study showing that a combination of dairy product fermented by lactobacilli and GOS improved calcium absorption in weaning rats [8]. In the preliminary study, we observed that in normal young adult rats, the YG diet increased calcium absorption and BMD of the whole femur compared with the control diet [21], suggesting that the YG diet has beneficial effects on calcium absorption and BMD regardless of PPI administration. Dietary GOS reportedly increased calcium absorption by the action of bacterial fermentation of GOS in the large intestine [13]. In addition, our previous study showed that caecal weight was larger in rats fed a combination of dairy product fermented by lactobacilli and GOS than in rats fed the dairy product fermented by lactobacilli or GOS alone, suggesting that dairy product fermented by lactobacilli and GOS additively or synergistically increased caecal fermentation. In the present study, we observed increased caecal weight in the PPI + YG group, indicating increased caecal fermentation [31,32], which could be implicated in the increased calcium absorption. The potential positive effects of low-digestible carbohydrates on calcium absorption in large intestine can be explained by the following mechanisms [33]. First, short-chain fatty acids (SCFAs) produced by the fermentation of low-digestible carbohydrates in large intestine decrease luminal pH, increasing calcium solubility [34]. Second, calcium may pass through the cell membrane of intestine more readily in the form of a lower-charge complex with SCFAs than in the ionized form [35,36,37]. Third, the fermentation is also accompanied by caecum enlargement, which may increase the intestinal absorption exchange area. The YG diet may also increase calcium absorption by these mechanisms, which appears to have beneficial effects on bone metabolism.

The YG diet partly inhibited the increase in phosphorus absorption in PPI-administered rats. Calcium is known to reduce phosphorus absorption [38,39], which suggests that the YG diet may inhibit the increase in phosphorus absorption in PPI-administered rats by increasing calcium absorption. The YG diet strongly suppressed serum 1,25(OH)_2_D levels and increased serum FGF23 levels. FGF23 decreases phosphorus absorption via inhibiting 1,25(OH)_2_D synthesis [26]. Therefore, the inhibitory effect of the YG diet on increased phosphorus absorption induced by PPI administration may also depend on the elevated FGF23 levels and subsequent decrease in 1,25(OH)_2_D levels. Rodriguez-Ortiz et al. [30] reported that an increase in dietary calcium increased FGF23 and decreased 1,25(OH)_2_D in parathyroidectomized rats. Therefore, increased calcium absorption by the YG diet may be involved in the increase in FGF23 levels and the decrease in 1,25(OH)_2_D levels. The present study showed that the YG diet increased calcium absorption regardless of low 1,25(OH)_2_D levels. Mineo et al. [40] reported that indigestible carbohydrates enhanced calcium absorption by activating the paracellular pathway, which is insensitive to vitamin D. These facts suggest that the YG diet may also activate the paracellular pathway of calcium absorption.

In weaning rats, PPI administration decreased calcium absorption, followed by the increase in bone resorption, leading to the decrease in BMD [7]. In contrast, PPI administration did not affect calcium absorption in adult rats, although it decreased BMD. Therefore, the mechanism by which PPI affected bone metabolism in adult rats remains to be clarified. However, a high phosphorus diet reportedly increased bone resorption via secondary hyperparathyroidism [41], while some studies reported that it influenced bone loss regardless of changes in calcium metabolism and PTH hypersecretion [42,43,44]. Therefore, it is possible that PPI administration induced bone loss via PTH-independent effects of high phosphorus status in adult rats and that the YG diet improved BMD, which may partly result from the improvement of high phosphorus status. Furthermore, we observed clear correlations between BMD of femur and serum FGF23 or 1,25(OH)_2_D levels in adult rats, which suggests that the changes in 1,25(OH)_2_D and FGF23 may be involved in bone metabolism. Tanabe et al. [45] suggested that milk and dairy products could improve bone metabolism in ovariectomized rats, at least partly through changes in 1,25(OH)_2_D and FGF23 levels, which supports our results.

The present study design did not include groups fed the diets containing yogurt or GOS alone in the absence or presence of PPI and a group fed the YG diet in the absence of PPI. Therefore, further studies are needed to investigate whether the effects of the YG diet in adult rats derived from the combination effects of yogurt and GOS or the single effects of yogurt or GOS and whether these effects depended on PPI administration. The inclusion of more experimental groups would also provide relevant additional information to explain the underlying mechanism and to define the most straightforward approach to overcome the adverse effects of PPI in terms of efficacy, price and commodity for patients. Furthermore, low-digestible carbohydrates at high doses were reported to induce dose-dependent diarrhea [46], which should also be considered in clinical application.

Our study has some limitations. First, because the pH of the cumulative gastric juice of animals receiving PPI + YG diet was not measured, we could not clarify the eventual contribution of this variable. Second, some medications were reported to bind to the divalent cations, resulting in poorly-absorbed complexes [47]. Therefore, we could not exclude the possibility that oral PPI administration might affect calcium absorption in adult rats, because PPI were subcutaneously administered in the present study.

## 5. Conclusions

Although PPI administration did not affect calcium absorption, it adversely affected BMD and influenced phosphorus metabolism in adult rats. Furthermore, the YG diet beneficially affected BMD and attenuated the effects of PPI administration on phosphorus metabolism.

## Figures and Tables

**Table 1:** Composition of the experimental diets.

	Control (AIN-93M)	Yogurt + GOS
Ingredients (g/kg diet)		
Corn starch	447.10	333.03
α-corn starch	155.00	155.00
Casein	140.00	46.67
Lyophilized yogurt	0.00	220.50
Sucrose	100.00	32.25
Soybean oil	40.00	40.00
Cellulose powder	50.00	50.00
AIN-93M mineral premix without calcium and phosphorus	35.00	35.00
Calcium carbonate	12.41	5.54
Potassium phosphate	8.69	2.46
AIN-93 vitamin premix including choline bitartrate	10.00	10.00
l-Cystine	1.80	1.80
GOS ingredient	0.00	67.75
Calculated value (g/kg diet)		
Crude protein	118.70	118.70
Calcium	5.00	5.00
Phosphorus	3.00	3.00
GOS	0.00	50.00

AIN, American Institute of Nutrition; GOS, galactooligosaccharides.
